# Dancing through neurocognitive changes: dance/movement therapy supporting caregivers and people living with Alzheimer's and other dementias

**DOI:** 10.3389/fpsyg.2024.1504559

**Published:** 2024-12-17

**Authors:** Cecilia Fontanesi, Donna Newman-Bluestein

**Affiliations:** ^1^Department of Dance and Biology, Barnard College, Columbia University, New York City, NY, United States; ^2^Department of Dance, Hunter College, City University of New York, New York City, NY, United States; ^3^Department Master of Arts Therapies, Codarts University of the Arts, Rotterdam, Netherlands; ^4^Dance for Connection, Boston, MA, United States

**Keywords:** major neurocognitive disorder, Alzheimer's, dementia, Dance/Movement Therapy (DMT), dance therapy, group therapy, caregiver

## 1 Introduction

A diagnosis of a progressive neurodegenerative disease implies a daily, personal loss that may be experienced by those directly affected by the condition and their caregivers. Dance/Movement Therapy (DMT) offers a complement to this perspective of what dementia is and what dementia does to people and personhood (Coaten and Newman-Bluestein, 2013; Newman-Bluestein, 2020).

Group and partnered dance sessions can support the emotional, relational, and physical needs of people living with major neurocognitive disorder^1^ (MND) and of those caring for them, not only supporting families keeping their members at home or in the community but also offering respite and emotional release for caregivers (Ruiz-Muelle and López-Rodríguez, 2019). DMT is particularly well-suited to supporting people with Alzheimer's Disease (AD) or MND, as it engages multiple sensory modalities, thus neural pathways and networks, and respects each individual's unique way of communicating and reconnecting with identity and personal narratives (Goldstein-Levitas, 2016).

People who have AD/MND frequently deal with a detachment from their memories, sense of self, and societal functions, as noted by Goldstein-Levitas (2016). These losses cause them to become isolated, confused, and emotionally distressed, affecting not just the individuals with AD/MND but also those caring for them (World Health Organization, 2012). DMT offers a pathway to reintegrate those fragmented aspects of the self through embodied expression (Hill, 2009; Newman-Bluestein, 2017). The familiarity of a tune, shared movements, or even a simple gesture can bring back emotions and memories in ways that language cannot, as Newman-Bluestein (2017) pointed out. Dance and movement may bridge the experiences of individuals with AD/MND and the efforts of the care providers to connect. In this article, we contemplate how the interactions within group DMT could offer mental and physical health benefits to these people, their families, and their caregivers.

## 2 Dance/movement therapy framework

DMT frameworks for working with people living with AD/MND necessarily embrace reflections on connection and empathy (Hill and Newman-Bluestein, 2010), embodiment (Coaten and Newman-Bluestein, 2013), non-verbal communication and relationship (Newman-Bluestein and Chang, 2017), and even dance aesthetics (Newman-Bluestein, 2020).

The following four concepts—*welcoming, shifting, present moment*, and *vitality*—are drawn as examples from Donna Newman-Bluestein's training manual for caregivers of people with AD/MND (Newman-Bluestein and Chang, 2017). These concepts illustrate how DMT practice can support this population, emphasizing approaches centered on both the needs of individuals with AD/MND and their relationships with their caregivers.

*Welcoming:* The DMT invites individuals to participate in a therapeutic space rooted in safety, dignity, respect for choice, and empowerment. Members are invited by name to participate actively and contribute to the group's space through their language, cultural background, musical preferences, and original contributions.

*Shifting:* The therapist observes even the smallest changes in the range of motion and movement patterns, in the spatial relationships between participants, in the expressive qualities of movement, and in the shapes of gestures, recognizing that these shifts may reflect changes in motivation, emotional investment, and mood. The therapist supports the development of a creative group atmosphere, understanding that the therapy process involves working together.

*Present moment:* Artistic creation through movement enhances present-centered awareness, allowing individuals to access a state of presence. The therapist creates a space that encourages participants to accept their current state, facilitating a process in which their relationships with time and embodiment surface, revealing what is most meaningful to them in the moment.

*Vitality:* Vitality may be described as the sensation of inhabiting one's body, influenced by different cultural viewpoints on body awareness, breath, and movement. Through dance, individuals are reminded that their bodies are alive and dynamic, and the therapist promotes a sense of being that aligns with each person's values and lived experiences.

## 3 Personhood, I-Thou relationship, and here-and-now interactions

When working with people with AD/MND, we find it essential to recognize their uniqueness and the possibility for meaningful connections, even when cognitive impairment is severe (Kitwood and Bredin, 1992). In the past, when defining personhood, the tendency has been to prioritize autonomy and logical thinking over feelings and relational potential (Kitwood, 1997a). Even with severe cognitive impairment, an I-Thou form of meeting and relating is usually attainable (Kitwood, 1997b). Drawing from Ich and Du, published in 1922 by Martin Buber, Kitwood refers to two ways of experiencing a relationship. The I-It mode implies coolness, detachment, instrumentality, maintaining distance, and avoiding risk. Conversely, the I-Thou mode requires individuals to reach out, be spontaneous, and embark on a journey into a shared space (Kitwood, 1997b, p. 10). This connection goes beyond the confines of language and cognition, tapping into the essence of our shared humanity. It highlights every individual's inherent worth and dignity, regardless of their cognitive abilities. This notion resonates with our belief that building compassionate relationships lies at the core of DMT.

Further, Kitwood's ideas parallel Yalom (2009)'s concept of the importance of here-and-now interactions within a group. The group becomes a social microcosm where individuals can connect by de-centralizing the therapist's position and facilitating spontaneous expression (Yalom, 2009). Yalom emphasizes fostering honesty and spontaneity of expression within the group. These qualities are the lifeblood that nurtures an authentic and vibrant group experience. Encouraging individuals to express themselves in the present moment allows meaningful connections to form, transcending the conventional boundaries of therapist-led interactions (Yalom and Molyn, 2005).

In this approach, the therapist's role shifts from being the primary focus to becoming a facilitator who carefully listens and observes. As group members interact and connect, the therapist's task is to relate the spontaneous offerings and concerns of individuals to the overarching goals of the group (Yalom, 2009). This relational exchange mirrors the diverse meanings and perspectives within each member's unique internal world (Yalom, 2009).

## 4 Exploring the benefits of group and partnership dynamics

During early stages, regular dance participation can improve physical and cognitive function, alleviate anxiety, and help reduce feelings of depression associated with AD/MND, such as falls and physical decline (Ruiz-Muelle and López-Rodríguez, 2019). From a mental health perspective, dance fosters emotional expression, alleviates anxiety, and helps reduce feelings of depression for both caregivers and individuals with AD/MND.

DMT groups that include caregivers and individuals with AD/MND create opportunities for relationship-building and mutual support, strengthening emotional bonds, and fostering resilience (Coaten and Newman-Bluestein, 2013). In a group setting, caregivers often feel empowered by collective energy and empathy, while individuals with AD/MND feel less isolated (Beardall et al., 2014).

Caregivers can engage with others experiencing comparable difficulties through DMT groups. This experience has the potential to alleviate feelings of aloneness and offer essential emotional encouragement. Champagne (2024) emphasizes that DMT can serve as a resilience-building tool for caregivers, fostering a sense of flexibility, positive emotion, and shared experience through movement. Research by Petts and Urmston (2022) demonstrates that participation in dance activities can reaffirm the caregiver-care receiver relationship, helping caregivers find respite, reconnect with their loved ones, and rediscover a sense of identity that caregiving often subsumes. In this setting, caregivers shift from being caretakers to partners, transforming their role into one of shared experience rather than solely obligation. This co-creation dynamic in DMT strengthens emotional bonds and allows participants to explore their relationships in a meaningful, non-verbal manner that fosters empathy, and closeness (Hill and Newman-Bluestein, 2010). In dance sessions, offering or following movements can create a shared experience that promotes emotional and social connectedness (Doe and Roe, 2023). This collective emotional space contributes to the participants' wellbeing, reducing stress and enhancing resilience in those with caregiving roles (Newman-Bluestein, 2017). For individuals with AD/MND, dance taps into neural pathways associated with memory, emotion, and movement. Familiar music and gestures can evoke thoughts, memories, and associations and bring moments of presence, even in those with severe cognitive impairments. However brief, these moments allow individuals and their caregivers to experience a deep sense of connection (Goldstein-Levitas, 2016).

## 5 Reflections on synchrony and syncopation in group and partnered DMT

Neuroscience increasingly supports the benefits of DMT, demonstrating that rhythmic movement can stimulate neural pathways involved in memory, emotion, and coordination, even in individuals with cognitive decline (Kshtriya et al., 2015). Studies indicate that synchronous movement enhances social bonding and influences neural and hormonal coupling, improving quality of life and wellbeing (Dieterich-Hartwell, 2024). Interpersonal synchrony (IS) fosters a unique relational experience, enabling participants to feel a sense of affiliation and closeness with others, which is especially beneficial for individuals with neurocognitive disorders and their caregivers.

Research on IS in children and adolescents highlights its impact on social bonding. For instance, Tunçgenç and Cohen (2016) suggested that synchronous movement may increase bonding and a sense of similarity among group members, even in groups with previous biases. Similarly, Prakash (2023) observed that synchronized movement in DMT can enhance social engagement, cooperation, and empathy. These findings support the idea that rhythmic synchrony may help bridge social divides and strengthen relational connections. In DMT, rhythm is a powerful mechanism for enhancing togetherness, group cohesion, and kinesthetic empathy. Prakash (2023) describes how rhythmic synchrony in group settings can create feelings of security and wellbeing as group members feed off each other's energy and rhythm.

Importantly, studies by Witek et al. (2014) and Matthews et al. (2019, 2020) reveal that syncopated rhythms and harmonic interplay activate brain regions associated with motor timing, reward, and the release of endorphins that foster social bonding. These results suggest that rhythmic complexity, rather than simple rhythmic synchrony, facilitates physical coordination and contributes to emotional engagement and joy, strengthening the bond between individuals in a group. Further, as Nelson et al. (2024) argued, relationships are not solely about precise synchrony. Moments of syncopation, or intentional rhythmic deviation, are critical in enhancing interactions' depth and relational richness. Unlike perfect synchrony, syncopation allows for “a relation between relations” rather than mere alignment, creating a dynamic space where differences and individuality emerge (Nelson et al., 2024). This “imperfect synchronization” can generate an aesthetic and therapeutic value, enriching the therapeutic process by allowing participants to experience empathy and connection without sacrificing their individuality. Syncopation thus enables participants to maintain personal expression while simultaneously engaging with others, allowing them to experience togetherness and difference.

In sum, synchrony and syncopation in DMT provide a multidimensional relational experience where rhythmic movement fosters emotional connection, empathy, and social bonding. By engaging in synchronous movement and navigating moments of syncopation, participants in DMT develop a sense of togetherness while preserving their identities. This nuanced dance of alignment and differentiation is central to the therapeutic impact of DMT, particularly for individuals with AD/MND and their caregivers, as it supports connection, personal expression, and mutual understanding in ways that transcend conventional therapeutic methods and language.

## 6 Conclusion

DMT offers a multidimensional approach to supporting individuals with AD/MND and their caregivers. It integrates elements of group support, meaningful relational dynamics, synchrony, and syncopation, fostering a shared sense of presence and connection. For individuals with AD/MND, these sessions create moments of coherence, helping them reclaim fragments of their identity and reconnect with their personal history and emotions. For caregivers, DMT provides a supportive space for shared experiences, transforming caregiving from an obligation into a meaningful partnership.

As virtual and in-person DMT frameworks evolve, the potential for broader access and positive impact on wellbeing expands. Future research should explore how collective meaning-making, synchrony and syncopation, and creative movement contribute to resilience, quality of life, and emotional expression in caregiving relationships, enriching the scope and application of DMT in neurocognitive care.

## Footnotes

^1^Dementia and major neurocognitive disorder (MND) are terms that describe a similar condition. However, we prefer to use the term MND instead of dementia, in line with the Diagnostic and Statistical Manual of Mental Disorders (DSM-5).
